# Clinical outcomes of EGFR-TKI treatment and genetic heterogeneity in lung adenocarcinoma patients with *EGFR* mutations on exons 19 and 21

**DOI:** 10.1186/s40880-016-0086-2

**Published:** 2016-03-21

**Authors:** Jiang-Yong Yu, Si-Fan Yu, Shu-Hang Wang, Hua Bai, Jun Zhao, Tong-Tong An, Jian-Chun Duan, Jie Wang

**Affiliations:** The Key Laboratory of Carcinogenesis and Translational Research (Ministry of Education); Department of Thoracic Medical Oncology, Peking University School of Oncology, Beijing Cancer Hospital and Institute, Beijing, 100142 P. R. China

**Keywords:** *EGFR* exon 19 deletion, *EGFR* exon 21 L858R point mutation, Lung adenocarcinoma, Treatment efficacy

## Abstract

**Background:**

Epidermal growth factor receptor (*EGFR*) mutations, including a known exon 19 deletion (19 del) and exon 21 L858R point mutation (L858R mutation), are strong predictors of the response to EGFR tyrosine kinase inhibitor (EGFR-TKI) treatment in lung adenocarcinoma. However, whether patients carrying *EGFR* 19 del and L858R mutations exhibit different responsiveness to EGFR-TKIs and what are the potential mechanism for this difference remain controversial. This study aimed to investigate the clinical outcomes of EGFR-TKI treatment in patients with *EGFR* 19 del and L858R mutations and explore the genetic heterogeneity of tumors with the two mutation subtypes.

**Methods:**

Of 1127 patients with advanced lung adenocarcinoma harboring *EGFR* 19 del or L858R mutations, 532 received EGFR-TKI treatment and were included in this study. *EGFR* 19 del and L858R mutations were detected by using denaturing high-performance liquid chromatography (DHPLC). T790M mutation, which is a common resistant mutation on exon 20 of *EGFR*, was detected by amplification refractory mutation system (ARMS). Next-generation sequencing (NGS) was used to explore the genetic heterogeneity of tumors with *EGFR* 19 del and L858R mutations.

**Results:**

Of the 532 patients, 319 (60.0%) had *EGFR* 19 del, and 213 (40.0%) had L858R mutations. The patients with *EGFR* 19 del presented a significantly higher overall response rate (ORR) for EGFR-TKI treatment (55.2% vs. 43.7%, *P* = 0.017) and had a longer progression-free survival (PFS) after first-line EGFR-TKI treatment (14.4 vs. 11.4 months, *P* = 0.034) compared with those with L858R mutations. However, no statistically significant difference in overall survival (OS) was observed between the two groups of patients. T790M mutation status was analyzed in 88 patients before EGFR-TKI treatment and 134 after EGFR-TKI treatment, and there was no significant difference in the co-existence of T790M mutation with *EGFR* 19 del and L858R mutations before EGFR-TKI treatment (5.6% vs. 8.8%, *P* = 0.554) or after treatment (24.4% vs. 35.4%, *P* = 0.176). In addition, 24 patients with *EGFR* 19 del and 19 with L858R mutations were analyzed by NGS, and no significant difference in the presence of multiple somatic mutations was observed between the two genotypes.

**Conclusions:**

Patients with *EGFR* 19 del exhibit longer PFS and higher ORR compared with those with L858R mutations. Whether the heterogeneity of tumors with *EGFR* 19 del and L858R mutations contribute to a therapeutic response difference needs further investigation.

## Background

The mutation frequency of epidermal growth factor receptor (*EGFR*) is approximately 35%–40% among Asian patients with stage IV non–small cell lung cancer (NSCLC) [1, 2]. Mutations are the most prevalent in East Asian, female, non-smoking patients with adenocarcinoma [3]. EGFR is involved in an important signal transduction pathway that regulates tumorigenesis and cell survival and is frequently overexpressed during the development and progression of NSCLC. Among the various types of *EGFR* mutations, the most common genetic alterations are in-frame deletions of exon 19 (19 del; approximately 44%), which encompasses the amino acids from codons L747 to E749, and the L858R point mutation of exon 21 (L858R mutation; approximately 41%) [4]. Notably, the tyrosine kinases with *EGFR* exon 19 del and L858R mutations exhibit a reduced affinity with adenosine triphosphate (ATP) but have a relatively high affinity with EGFR tyrosine kinase inhibitors (EGFR-TKIs) and, therefore, generate an antitumor effect [5, 6]. *EGFR* mutation status is the most crucial factor for NSCLC patients in the clinical response to EGFR-TKIs [6]. A series of phase III randomized-controlled trials (RCTs) have shown that patients with *EGFR*-mutated NSCLC who received EGFR-TKI treatment had a higher objective response rate (ORR), longer progression-free survival (PFS), and better quality of life (QoL) than those who received standard chemotherapy [7–9]. However, these studies did not report an overall survival (OS) benefit of EGFR-TKI therapy in NSCLC patients.

Recently, a pooled analysis of two multicenter randomized clinical studies (LUX-Lung 3 and LUX-Lung 6) compared first-line chemotherapy in patients who carried *EGFR* mutations with afatinib, a second-generation, irreversible EGFR-TKI [10]. The results showed that patients with *EGFR* 19 del who received afatinib treatment had a significantly longer OS compared with those treated with platinum-based chemotherapy. On the contrary, patients with L858R mutations presented longer OS in the chemotherapy group than in the afatinib treatment group, although the difference did not reach statistical significance. Thus, the researchers concluded that the tumors with *EGFR* 19 del and L858R mutations can be thought of as two different diseases that require different treatment strategies. This conclusion generated great controversy regarding the following points: (1) whether the tumors with *EGFR* 19 del and L858R mutations are indeed two different diseases; (2) whether first-generation EGFR-TKIs can achieve the same results as afatinib in patients who possess the *EGFR* 19 del or L858R mutations; and (3) whether the genetic heterogeneity of the NSCLC patients with the two genotypes is associated with different clinical responses to EGFR-TKIs. Providing answers to these controversies or questions would help optimize the individualized treatment strategies for advanced NSCLC.

Here, we retrospectively analyzed the efficacy of EGFR-TKI therapy on metastatic NSCLC with an *EGFR* 19 del or an L858R mutation. Given the co-existence of uncommon mutations of *EGFR* including T790M mutation and other gene mutations might influence the efficacy of EGFR-TKI between these two sensitive groups [11, 12], we deeply explored the difference in heterogeneity between tumors with the two *EGFR* mutation subtypes.

## Population and methods

### Patient population

Among 1127 patients with histologically confirmed lung adenocarcinoma (stage IIIB or IV) possessing either the *EGFR* 19 del or L858R mutation treated at the Peking University Cancer Hospital between April 2004 and September 2014, 532 patients treated with EGFR-TKIs were included in this study. The objective response was assessed according to the response evaluation criteria in solid tumors (RECIST) 1.1 criteria [13]. Patients without measurable lesions according to the RECIST 1.1 criteria were excluded. Informed consent to allow the use of biopsy tissue for genetic analyses was obtained from all patients. This study was reviewed and approved by the Institutional Ethics Committee of Peking University Cancer Hospital.

Patient characteristics were determined by a retrospective chart review, including age at diagnosis, sex, smoking status, clinical stage, and Eastern Cooperative Oncology Group (ECOG) performance status (PS) at the initial treatment with EGFR-TKI and chemotherapy. Smoking status was based on records at the patients’ first clinic visit; smokers were defined as having smoked more than 100 cigarettes in a lifetime. All patients with *EGFR* mutations were recommended to receive gefitinib (250 mg daily), erlotinib (150 mg daily), or icotinib (375 mg daily) according to individual preference until disease progression, unacceptable toxicities, or self-withdrawal.

### *EGFR* mutation evaluation

Tumor tissues for *EGFR* analysis were collected at the time of diagnosis or recurrence before receiving EGFR-TKI therapy. The *EGFR* 19 del or L858R mutation was detected by using denaturing high-performance liquid chromatography (DHPLC) [14]. The T790M mutation was detected with an amplification refractory mutation system (ARMS) [15].

### Sample collection, library preparation, and next-generation sequencing

To find possible gene alterations that account for the potential mechanism resulting in differences in clinical outcome between the *EGFR* 19 del and L858R mutation groups, we selected 12 patients with the *EGFR* 19 del (seven with paired normal leukocytes and five without) and another 12 patients with the L858R mutation (nine with paired normal leukocytes and three without) via simple random sampling. Samples of the 12 patient pairs above were used for next-generation sequencing (NGS) to detect a panel of 483 cancer-related genes, including all sites of *EGFR* mutations, other known driver genes, drug targets, and drug-resistant genes. Another 19 patients who were identified from a Novogene Company database, including 12 patients with *EGFR* 19 del and 7 with L858R mutations, were also analyzed. Genomic DNA was extracted by using the Qiagen blood mini kit (Qiagen, Hilden, Germany) according to the manufacturer’s instructions. DNA libraries were prepared using the NEBNext DNA Library Prep Reagent Set (New England BioLabs, Ipswich, MA, USA). All exons of the 483 cancer-related genes and 88 introns from 14 genes that are frequently rearranged in cancer were captured using Agilent SureSelect XT (Agilent, Santa Clara, CA, USA). The libraries were sequenced using paired-end 150-bp reads on a Hiseq sequencing system (Illumina, Beijing, China). The median sequencing depth was 469× per sample (ranging from 38× to 3883×).

### Statistical analysis

The Wilcoxon rank-sum test or χ^2^ test was used to test the difference of clinical and pathologic parameters between patients with the *EGFR* 19 del and L858R mutations. The clinical response to EGFR-TKI treatment was evaluated based on computed tomography (CT) scans every 2 months during treatment and was classified as complete response (CR), partial response (PR), stable disease (SD), or progressive disease (PD) by using the standard RECIST 1.1 criteria. The ORR and disease-control rate (DCR) between patients with the two mutation types were calculated and compared by using χ^2^ tests. PFS was calculated from the start of EGFR-TKI therapy to treatment failure (disease progression, death, or appearance of unacceptable toxicity) or the date of the last follow-up. OS was defined as the first day of EGFR-TKI therapy until death from any cause or the date of the last follow-up. Survival curves were estimated by using the Kaplan–Meier method, and the differences between groups were analyzed by using the log-rank test. The multiple Cox’s proportional hazard model was used for univariate and multivariate analyses to assess the variables including age, sex, smoking status, PS status, *EGFR* mutation type, EGFR-TKI drugs, and treatment lines of TKI therapy and to predict the hazard rates for PFS and OS. The Fisher’s exact test was used to select the different mutations between the *EGFR* 19 del and L858R mutation single nucleotide variation (SNV) samples. Genes with a significant difference in expression between the two groups were further analyzed. Lastly, mutation frequencies were used to determine whether the mutations were somatic or germline mutations. The statistical significance level was defined as two-sided *P* < 0.05. All statistical analyses were performed with the SPSS statistical software, version 19.0 (IBM Corp., Armonk, NY, USA).

## Results

### Patient characteristics

Of the 532 patients with an *EGFR* mutation, 319 (60.0%) harbored an *EGFR* 19 del, and 213 (40.0%) had the L858R mutation. The patients’ clinical characteristics are shown in Table 1. The median age of the patients was 59 years (range, 20–86 years); the majority of patients were women (60.5%) and non-smokers (68.6%). There were 369 patients (69.4%) who received gefitinib, 82 patients (15.4%) who received erlotinib, 56 patients (10.5%) who received icotinib, and 25 patients (4.7%) who could not be verified. All 532 NSCLC patients were treated with EGFR-TKIs; among these patients, 280 (52.6%) received EGFR-TKIs as a first-line therapy, 233 (43.8%) as a second-line therapy or greater, 11 (2.1%) as a maintenance therapy, and 8 (1.5%) of which could not be verified in this regard.

**Table 1:** Baseline characteristics of 532 patients with non–small cell lung cancer (NSCLC)

Characteristic	No. of patients	Exon 19 deletion	Exon 21 L858R mutation	*P* value^b^
Total	532	319	213	
Age^a^ (years)				0.001
Median	59	57	61	
Range	20–86	20–86	31–81	
Sex				0.068
Male	210	136	74	
Female	322	183	139	
Smoking status				0.931
Never smoker	365	220	145	
Ever smoker	145	88	57	
Unknown	22	11	11	
ECOG PS				0.202
0	117	58	59	
1	292	178	114	
2	26	15	11	
3	8	4	4	
Not evaluated	89	64	25	
Clinical stage				0.572
IIIB	28	14	14	
IV	495	301	194	
Unknown	9	4	5	
EGFR-TKI				0.270
Gefitinib	369	210	159	
Erlotinib	82	56	26	
Icotinib	56	34	22	
Unknown	25	19	6	
TKI line				0.004
Maintenance	11	1	10	
Line 1	280	157	123	
Line 2	173	118	55	
Line ≥3	60	36	24	
Unknown	8	7	1	

*ECOG* Eastern Cooperative Oncology Group, *PS* performance status, *EGFR* epidermal growth factor receptor, *TKI* tyrosine kinase inhibitor

^a^Except for this value, other values are all presented as the number of patients

^b^Analyzed by using χ^2^ test

### Objective response

Of the 532 patients, 79 were excluded due to the lack of response evaluation, and the other 453 were divided into two groups according to the *EGFR* 19 del and L858R mutation statuses (Table 2). The clinical responses, including CR, PR, SD, and PD, did not significantly differ between the two groups (*P* = 0.074). For the whole cohort, the ORR of TKIs in patients with the *EGFR* 19 del was significantly higher than that in patients with the L858R mutation (55.2% vs. 43.7%, *P* = 0.017). There was no significant difference in the DCR between 19 del and L858R mutation groups (94.1% vs. 89.6%, *P* = 0.081). In the stratified analysis, the *EGFR* 19 del group displayed higher ORR to gefitinib than the L858R mutation group (59.2% vs. 43.2%, *P* = 0.005). However, no significant differences were observed in patients treated with erlotinib (55.8% vs. 39.1%, *P* = 0.184) or icotinib (33.3% vs. 58.8%, *P* = 0.089).

**Table 2:** Response to EGFR-TKIs in 453 NSCLC patients with different *EGFR* genotypes

Variate	Exon 19 deletion (*n* = 270)	Exon 21 L858R mutation (*n* = 183)	*P* value^a^
Best response (cases)			0.074
CR	4	2	
PR	145	78	
SD	105	84	
PD	16	19	
MDT (months)			
CR/PR	13.0	14.6	0.874
SD	13.7	11.9	0.193
ORR (%)			
Total	55.2	43.7	0.017
Line 1	60.0	49.5	0.108
Line ≥ 2	49.6	32.4	0.018
Gefitinib	59.2	43.2	0.005
Erlotinib	55.8	39.1	0.184
Icotinib	33.3	58.8	0.089
DCR (%)			
Total	94.1	89.6	0.081
Line 1	96.2	92.4	0.208
Line ≥2	91.7	84.5	0.113

*CR* complete response, *PR* partial response, *SD* stable disease, *PD* progressive disease, *ORR* objective response rate, *ORR* = CR + PR, *DCR* disease control rate, *DCR* = CR + PR + SD, *MDT* median duration of treatment, *ECOG* Eastern Cooperative Oncology Group, *PS* performance status, *EGFR* epidermal growth factor receptor, *TKI* tyrosine kinase inhibitor

^a^Analyzed by using χ^2^ test

### Progression-free survival

**Fig. 1:**
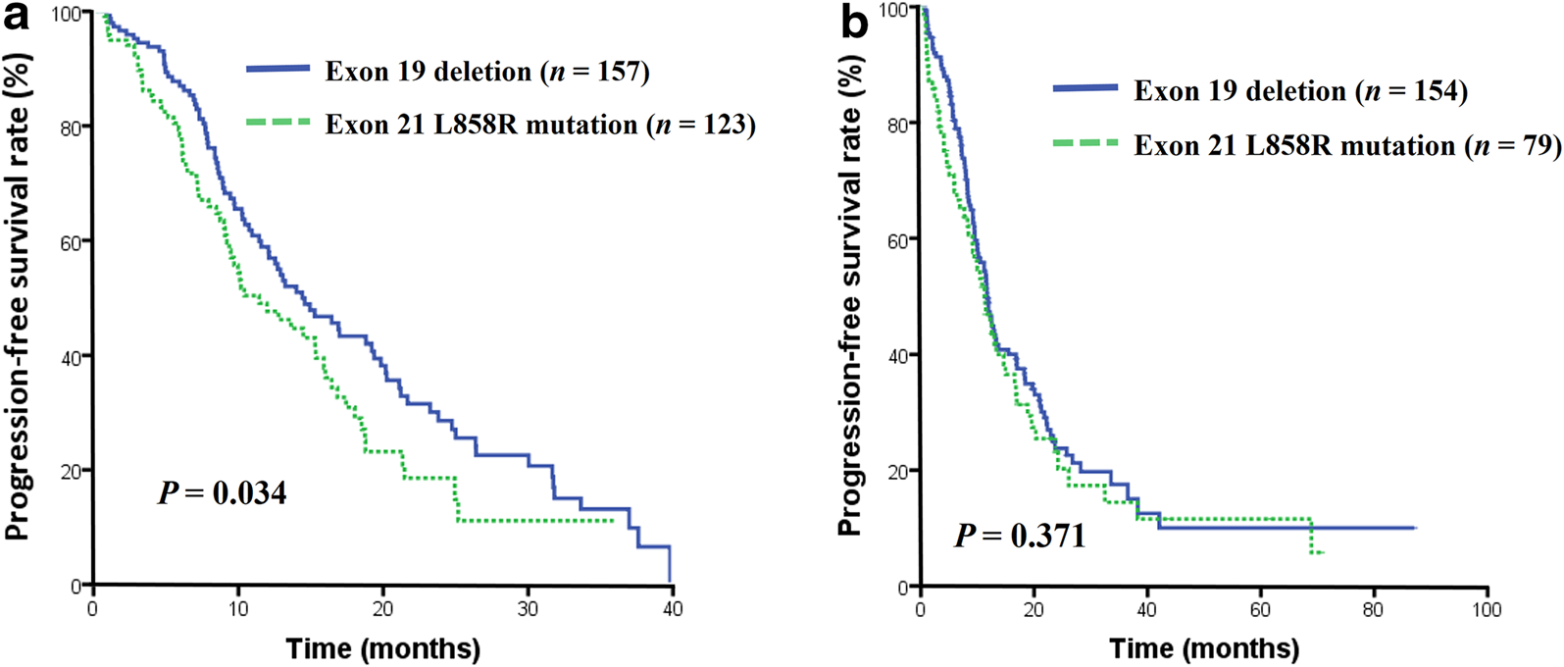
Progression-free survival (*PFS*) curves of epidermal growth factor receptor (*EGFR*)-mutated patients treated with tyrosine kinase inhibitors (*TKIs*). **a** PFS curves of patients with the *EGFR* exon 19 deletion (19 del) and exon 21 L858R point mutation (L858R mutation) who received first-line TKI therapy (14.4 vs. 11.4 months, *P* = 0.034); **b** PFS curves of patients with the *EGFR* 19 del and L858R mutation who received second-line or greater TKI therapy (11.7 vs. 11.2 months, *P* = 0.371)

Up to the last follow-up in September 2014, the median PFS in the entire cohort was 12.5 months (95% confidence interval [CI], 11.2–13.7 months). In patients treated with a TKI as the first-line therapy, the patients with *EGFR* 19 del had significantly longer PFS compared with those with L858R mutation (14.4 vs. 11.4 months, *P* = 0.034; Fig. 1a). There was no significant difference in PFS between the two groups with TKI as second-line therapy or greater (11.7 vs. 11.2 months, *P* = 0.371; Fig. 1b).

We subsequently analyzed whether there was a difference in PFS between patients with the same mutation subtype (the *EGFR* 19 del or L858R mutation) receiving different EGFR-TKI agents. No significant differences were observed between every two agents. Further, we investigated whether the same EGFR-TKI agent has different effects on patients with different mutation subtypes. In the first-line therapeutic setting of EGFR-TKIs, gefitinib could provide the patients harboring *EGFR* 19 del with a significantly longer PFS compared with those carrying L858R mutations (13.2 vs. 10.0 months, *P* = 0.008). In addition, this trend of prolonged PFS was also observed in the subgroup that received erlotinib as a first-line therapy, although the difference did not reach statistical significance (19.4 vs. 11.4 months, *P* = 0.889).

Stepwise Cox proportional hazards analysis was carried out to evaluate the association between PFS and the clinical characteristics described above. For patients with *EGFR* mutations who received TKI as a first-line therapy, both univariate and multivariate analysis showed that *EGFR* mutation status was the only predictive factor for PFS (*P* = 0.035 and *P* = 0.017, respectively), as shown in Table 3.

**Table 3:** Univariate and multivariate analyses for the PFS and OS of patients with tissue detected mutation and the use of TKIs as first-line therapy

Variable	PFS				OS			
	Univariate		Multivariate		Univariate		Multivariate	
	HR (95% CI)	*P* value^a^	HR (95% CI)	*P* value^a^	HR (95% CI)	*P* value^a^	HR (95% CI)	*P* value^a^
Age	1.000 (0.986–1.014)	0.980	0.993 (0.977–1.009)	0.381	1.008 (0.987–1.029)	0.480	0.996 (0.973–1.020)	0.757
Sex (men vs. women)	1.149 (0.830–1.590)	0.404	1.520 (0.916–2.522)	0.105	1.540 (0.992–2.393)	0.055	1.776 (0.890–3.544)	0.103
Smoking status (ever vs. never)	0.907 (0.628–1.311)	0.604	0.616 (0.363–1.044)	0.072	1.299 (0.807–2.091)	0.282	0.835 (0.405–1.722)	0.626
ECOG PS (2 vs. 0-1)	0.969 (0.523–1.796)	0.921	1.019 (0.536–1.935)	0.955	1.054 (0.454–2.447)	0.903	1.281 (0.531–3.091)	0.582
EGFR-TKI (erlotinib vs. gefitinib)	0.828 (0.531–1.291)	0.405	0.765 (0.448–1.307)	0.327	1.862 (0.581–5.962)	0.295	0.756 (0.357–1.603)	0.466
EGFR-TKI (icotinib vs. gefitinib)	0.652 (0.359–1.184)	0.160	0.702 (0.362–1.361)	0.295	1.917 (0.551–6.664)	0.306	0.255 (0.063–1.037)	0.056
Stage (IIIB vs. IV)	0.552 (0.203–1.498)	0.243	0.402 (0.119–1.356)	0.142	0.878 (0.276–2.795)	0.826	0.600 (0.144–2.506)	0.483
*EGFR* mutation (exon 19 del vs. L858R mutation)	0.705 (0.509–0.976)	0.035	0.631 (0.432–0.920)	0.017	0.878 (0.563–1.370)	0.566	0.671 (0.397–1.133)	0.135

*PFS* progression-free survival, *OS* overall survival, *HR* hazard ratio, 95% *CI* 95% confidence interval, *ECOG* Eastern Cooperative Oncology Group, *PS* performance status, *EGFR* epidermal growth factor receptor, *TKI* tyrosine kinase inhibitor

^a^Analyzed by using univariate and multivariate COX regression adjusted for age, sex, smoking status, ECOG PS, EGFR-TKIs, and stage

### Overall survival analysis

**Fig. 2:**
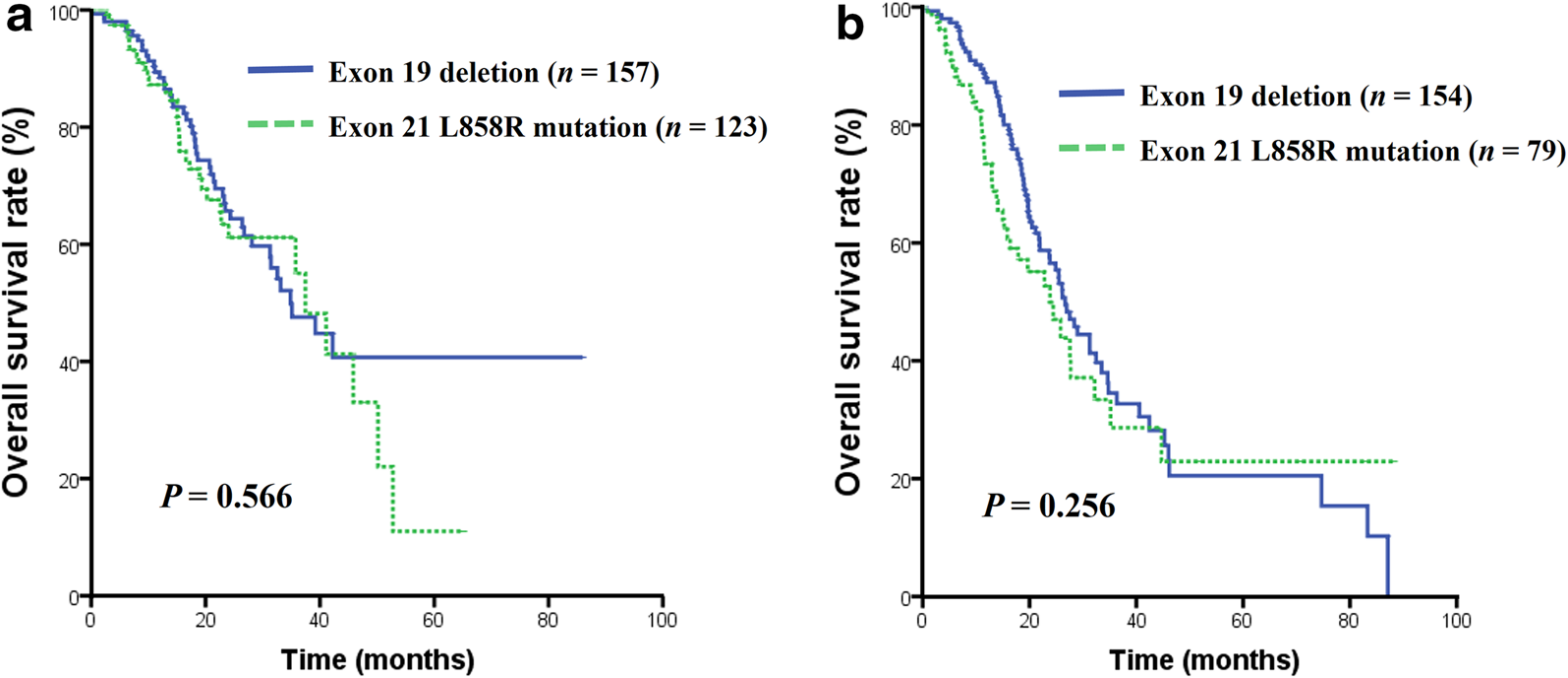
Overall survival (*OS*) curves of *EGFR*-mutated patients treated with TKIs. **a** OS curves of patients with *EGFR* 19 del and L858R mutation who received first-line TKI therapy (34.9 vs. 37.5 months, *P* = 0.566); **b** OS curves of patients with the *EGFR* 19 del and L858R mutation who received second-line or greater TKI therapy (26.7 vs. 23.8 months, *P* = 0.256)

The median OS for the entire cohort was 31.3 months (95% CI, 26.8–35.7 months). There was no significant difference in median OS between the *EGFR* 19 del and L858R mutation groups with first-line TKI treatment (34.9 vs. 37.5 months, *P* = 0.566; Fig. 2a) or second-line or greater TKI treatment (26.7 vs. 23.8 months, *P* = 0.256; Fig. 2b). Multivariate analysis did not reveal any predictive factor for OS, as shown in Table 3.

### Association of the *EGFR* mutations with the T790M mutation

The T790M mutation status is shown in Table 4 according to different *EGFR* genotypes and TKI therapy. The frequency of the T790M mutation in the post-EGFR-TKI treatment group was significantly higher than that in the pre-EGFR-TKI treatment group (28.4% vs. 6.8%, *P* < 0.001), and the co-existence of T790M mutation with *EGFR* 19 del in pre- and post-EGFR-TKI treatment groups was lower than that with the L858R mutation, but this difference was not statistically significant (5.6% vs. 8.8%, *P* = 0.554; 24.4% vs. 35.4%, *P* = 0.176, respectively).

**Table 4:** Difference of co-existence of T790M mutation between patients with *EGFR* 19 del and L858R mutation

Patient	Exon 19 deletion	Exon 21 L858R mutation	*P* value^a^
	(*n* = 140)	(*n* = 82)	
T790M mutation pre-TKI			0.554
Positive	3 (5.6)	3 (8.8)	
Negative	51 (94.4)	31 (91.2)	
T790M mutation post-TKI			0.176
Positive	21 (24.4)	17 (35.4)	
Negative	65 (75.6)	31 (64.6)	

^a^By using χ^2^ test. All values are presented as the number of patients followed by the percentage in the parentheses

### Association of the *EGFR* mutations with the multi-genes aberrances

**Fig. 3:**
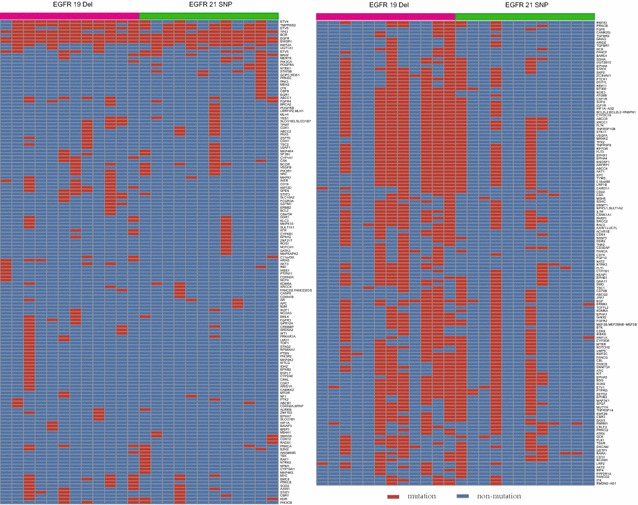
Heat map of 483 cancer-related genes in 12 pairs of patients with the *EGFR* 19 del or L858R mutation. The *red squares* represent genes with a mutation, and the *blue squares* represent those with no mutation

**Fig. 4:**
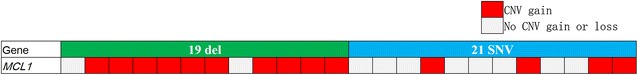
Copy number variation (CNV) of myeloid cell leukemia sequence 1 (*MCL1*) in 12 pairs of patients with the *EGFR* 19 del or L858R mutation. The *red squares* represent the gain of *MCL1* CNV. The *blank squares* represent no gain or loss of *MCL1* CNV

In total, 43 patients were involved in our genetic heterogeneity analysis, and there was no significant difference in the distribution of 483 cancer-related genes between the *EGFR* 19 del and L858R mutation groups. Twelve patient pairs from our center with relatively complete clinical information were further analyzed (Fig. 3). There were 20 different SNV/InDels (insertions/deletions) identified in the tumor tissue samples from the 12 patient pairs with an *EGFR* 19 del or L858R mutation (*P* < 0.05; Table 5). Except for *EGFR* mutations, the mutation frequency for the rest 19 differential SNV/InDels approximately equaled 50% or 100%, and these SNV/InDels were found in the tumor tissues of patients whose corresponding leukocytes were not sequenced due to the deficiency of the samples. Considering the genetic constitution of diploid organisms, we believed that these mutations were germline mutations rather than somatic ones. Therefore, the NGS analysis of the small samples did not display the difference in the somatic mutations between the two genotypes. The copy number variation of myeloid cell leukemia sequence 1 (*MCL1*) occurs more frequently in patients with an *EGFR* 19 del (10 of 12 patients) compared with those with the L858R mutation (4 of 12 patients); this difference was significant (*P* = 0.040; Fig. 4).

**Table 5:** List of genes differentially expressed between the *EGFR* exon 19 deletion and exon 21 L858R mutation groups

Gene	Chromosome	Reference base	Mutated base	Mutation frequency			*P* value^a^
				*EGFR* 19 deletion	Exon 21 L858R mutation	Difference	
*EGFR*	7	T	–	0	1	1	0.000
*PARP1*	1	A	G	0.583	0.083	0.5	0.027
*SPEN*	1	T	C	0.417	0	0.417	0.037
*SPEN*	1	A	G	0.417	0	0.417	0.037
*IGF1R*	15	GGT	G	0.417	0	0.417	0.037
*NOS3*	7	C	G	0.583	0	0.583	0.005
*KMT2D*	12	G	A	0.417	0	0.417	0.037
*IGF1R*	15	G	A	0.417	0	0.417	0.037
*SUFU*	10	G	A	0.583	0.083	0.5	0.027
*EP300*	22	T	C	0.583	0.083	0.5	0.027
*SPEN*	1	A	G	0.417	0	0.417	0.037
*ACVR1B*	12	C	T	0.417	0	0.417	0.037
*FLT1*	13	T	C	0.417	0	0.417	0.037
*IGF1R*	15	T	C	0.417	0	0.417	0.037
*RARA*	17	C	T	0.417	0	0.417	0.037
*CSF1R*	5	G	A	0.417	0	0.417	0.037
*ZC3HAV1*	7	G	A	0.417	0	0.417	0.037
*NOS3*	7	T	G	0.5	0	0.5	0.014
*ATG9B*	7	G	T	0.583	0.083	0.5	0.027
*PARP1*	1	C	G	0.583	0.083	0.5	0.027

^a^Analyzed by using Fisher’s exact test

## Discussion

The current study retrospectively investigated the clinical outcomes of 532 lung adenocarcinoma patients harboring *EGFR* 19 del or L858R mutation after first-generation EGFR-TKI treatment. The ORR was significantly higher in the *EGFR* 19 del group than in the L858R mutation group. When EGFR-TKI was used as a first-line treatment, the patients with the *EGFR* 19 del presented significantly longer PFS compared with those with the L858R mutation. However, no significant difference of OS between the two mutated subgroups was observed. Either gefitinib or erlotinib therapy provided the patients carrying an *EGFR* 19 del with a longer PFS than those with L858R mutations, although the difference in the erlotinib group did not reach statistical significance. These results suggested that PFS differences in the response to EGFR-TKI therapy between the patients with *EGFR* 19 del and L858R mutations may mainly derive from gefitinib and erlotinib therapy. However, this theory requires addition studies for confirmation.

The NGS technique was used in 43 patients with the *EGFR* 19 del or L858R mutation for genetic heterogeneity analysis. We found that there was no difference in uncommon *EGFR* mutations or other somatic mutations between the two mutation subtypes. However, according to our data and those of other investigators, patients with the *EGFR* 19 del indeed exhibited a longer PFS than patients with the L858R mutation [7–9]. Possible reasons resulting in the difference in PFS were speculated to be related to the space structure, different drug affinity with EGFR-TKIs, and genetic heterogeneity between these two genotypes [16, 17].

First, just like the tyrosine kinases of sensitive *EGFR* mutations exhibit a relatively higher affinity with EGFR-TKIs compared with the affinity with ATP, *EGFR* 19 del might be efficiently inhibited by EGFR-TKIs compared with the L858R mutation [5]. We assume that EGFR structural alterations caused by the *EGFR* 19 del may lead to a tighter combination with EGFR-TKIs compared with those changes caused by the L858R mutation. However, in vitro studies have demonstrated that NSCLC cell lines that possess the *EGFR* 19 del and L858R mutation had a similar degree of EGFR phosphorylation and almost equally growth inhibited by equivalent concentration of gefitinib [18, 19]. As a consequence, it is now unknown whether different conformations and/or affinities with EGFR-TKIs between *EGFR* 19 del and L858R mutations provide an explanation for the difference in survival. These issues still need further study.

Second, the influence of genetic heterogeneity in *EGFR*-mutated tumors on the response to EGFR-TKI treatment has been confirmed by several studies. Previous studies have reported that the T790M mutation, which is associated with an acquired resistance to reversible EGFR-TKIs [20, 21], might occur more frequently in patients harboring the L858R mutation than in those with an *EGFR* 19 del [11]. Furthermore, the co-existence of *EGFR* 19 del or L858R mutations with other mutations might influence the sensitivity to EGFR-TKIs [22–24]. Hata et al. [12] investigated the frequency of multiple drug-sensitive and drug-resistant mutations related with EGFR-TKIs in 783 NSCLC patients and found that eight patients who carried overlapping G719S and L858R mutations presented short PFS and a low ORR in response to gefitinib. In our study, we performed NGS, which included a panel of 483 cancer-related genes in 43 patients using tissue samples that contained *EGFR* exon 18–21 mutations. The results of NGS showed no significant difference in the presence of uncommon *EGFR* mutations or other somatic mutations between the two genotypes. However, due to the small sample size for NGS analysis in the current study, we cannot make a conclusion that genetic heterogeneity of tumors with *EGFR* 19 del or L858R mutations is not associated with differences in clinical response to EGFR-TKIs for the patients with the two genotypes. A larger sample size should be used and/or a prospective study should be conducted for further validation.

We also found that the appearance of *MCL1* copy number variation was more frequent in patients with *EGFR* 19 del compared with those harboring the L858R mutation (10/12 vs. 4/12, *P* = 0.04). *MCL1* gene expression was thought to be significantly associated with chemo- and radio-resistance and poor prognosis among NSCLC patients [25]. In contrast, several studies have indicated that MCL1 overexpression was a protective factor against breast cancer and can reduce tumor cell proliferation and arrest cell cycle progression [26]. Due to the small sample size of patients and insufficient sequencing depth in the present study, we cannot draw a sound conclusion that *MCL1* contributes to the different outcomes of the patients with two distinct genotypes. Although the panel used in our study covered 483 genes, there exists a possibility that other key co-existing genetic alterations are not included in this panel. Therefore, genetic profiling on a larger scale, such as whole exon sequencing or whole genome sequencing, should be performed for the further analysis of other genes that may influence the different outcomes of the two genotypes.

In addition, our results indicated that the OS was similar in two subtype groups of patients treated with first-generation EGFR-TKIs, but significant difference in OS was observed in patients treated with the second-generation EGFR-TKI afatinib in a previous study [10]. Unlike first-generation EGFR-TKIs, the second-generation EGFR-TKI afatinib is an irreversible inhibitor of EGFR and epidermal receptor 2 (Her-2) tyrosine kinase. Afatinib not only targets EGFR but also has an inhibitory effect on Her-2 [10]. The different drug targets may also contribute to differences in OS between patients treated with first- and second-generation EGFR-TKIs.

In conclusion, for patients with advanced lung adenocarcinoma, the use of first-line EGFR-TKIs in patients who harbored the *EGFR* 19 del might be associated with higher ORR and longer PFS compared with patients who carried the L858R mutation. Regardless of the use of EGFR-TKIs as a first-line, second-line, or greater treatment, there was no significant difference in the OS between the two mutation subgroups. We also found that there was no difference in genetic heterogeneity between these two mutation subtypes using the NGS technique.
